# Structural Characterization and Immunomodulatory Activity of Fructan Polysaccharides from Two Varieties of *Codonopsis pilosulae* (*C. pilosula* Nannf. var. *modesta* and *C. pilosula* (Franch.) Nannf.)

**DOI:** 10.3390/foods15030495

**Published:** 2026-02-01

**Authors:** Jingjing Dong, Xue Bai, Ziyang Wu, Xinxin Ma, Xiaoping Jin, Bei Fan, Fengzhong Wang, Jing Sun

**Affiliations:** 1 School of Food Science and Engineering, Shanxi Agricultural University, Jinzhong 030801, China; 15935656726@163.com; 2 Laboratory of Quality and Safety Risk Assessment on Agro-Products Processing, Key Laboratory of Agro-products Quality and Safety Control in Storage and Transport Process, Ministry of Agriculture and Rural Affairs, Institute of Food Science and Technology, Chinese Academy of Agricultural Sciences, Beijing 100193, China; baixue02@caas.cn (X.B.); wuziyang@caas.cn (Z.W.); m18245202569@outlook.com (X.M.); wangfengzhong@sina.com (F.W.); 3 Gannan Zhongzang Pharmaceutical Co., Ltd., Gannan Tibetan Autonomous Prefecture, Hezuo 746300, China; jinxiaoping2025@163.com

**Keywords:** *Codonopsis pilosula*, polysaccharides, structural characterization, immunomodulation

## Abstract

Although *Codonopsis pilosula* (common name: Dangshen) is a widely used medicinal edible herb, but the structural divergence and functional specificity of polysaccharides from its intraspecific varieties remain understudied. Therefore, this study explores the structural characteristics and immunomodulatory activities of polysaccharides from two varieties of *Codonopsis pilosula*, namely, *Codonopsis pilosula* Nannf. var. *modesta* (Nannf.) L.T.Shen and *Codonopsis pilosula* (Franch.) Nannf. Crude polysaccharides were extracted via water extraction–ethanol precipitation and purified using DEAE-52 column chromatography to obtain homogeneous fractions WCP (6.444 kDa) and BCP (15.71 kDa). Structural analyses confirmed both to be fructans, but with distinct linkage patterns: WCP contains Fru*f*-(2→, Glc*p*-(1→, →1)-Fru*f*-(2→, →1,6)-Fru*f*-(2→ linkages, while BCP additionally harbors →6)-Fru*f*-(2→ linkages. This structural divergence correlates with the observed differences in their physical properties and biological activities. Using zebrafish models, WCP and BCP were found to increase the density of neutrophils and macrophages, while reducing the levels of pro-inflammatory cytokines including IL-1β, IL-6, and TNF-α (*p* < 0.05 vs. model group). These results support their immunomodulatory potential, providing a scientific basis for developing *Codonopsis* polysaccharide-based functional food ingredients.

## 1. Introduction

*Codonopsis pilosula* (CP), commonly called Dangshen in China, is a widely recognized perennial herb valued for both its medicinal and culinary uses and is predominantly distributed throughout East, Southeast, and Central Asia [1]. The genus *Codonopsis* comprises over 49 species, with China serving as the primary production area, hosting approximately 39 species [1]. CP contains a variety of bioactive components—primarily polysaccharides, flavonoids, alkaloids, and steroids—which contribute to its diverse pharmacological properties, including immunomodulatory, anti-inflammatory, hypoglycemic, antiviral, anticancer, antioxidant, gastroprotective, and neuroprotective effects [2]. Apart from being used in medicine, CP is also widely used as a dietary supplement in tea, wine, soup, porridge pastes, and dishes such as hot pot [3].

The quality and market value of CP vary significantly across its varieties [1], with notable examples including *Codonopsis pilosula* Nannf. var. *modesta* (Nannf.) L.T.Shen (WD) and *Codonopsis pilosula* (Franch.) Nannf. (BTD). Different CP varieties exhibit considerable variations in phenotype, quality, and efficacy, potentially influencing differences in their chemical composition and biological activity [4]. Research to date on such differences among various CP varieties remains limited [5], representing an important research direction for advancing in-depth understanding and promoting the high-quality development of CP.

In recent years, the correlations between polysaccharide structural characteristics and biological activity have become a research hotspot, with their biological activities having been shown to be influenced by structural parameters such as monosaccharide composition, molecular weight, and glycosidic linkage patterns, endowing them with a range of unique biological properties [6]. As a major class of bioactive components in CP, polysaccharides exhibit a broad spectrum of biological activities, including immunomodulatory, anti-inflammatory, and antioxidant effects [7]. They have also demonstrated potential therapeutic value in treating conditions such as peptic ulcers, diabetes [8], and Alzheimer’s disease [9]. Nevertheless, comparative investigations focused on the fine structural characteristics of polysaccharides derived from WD and BTD, as well as their respective immunomodulatory activities, remain scarce.

Immunomodulation represents one of the core pharmacological effects of CP. Existing research has indicated that these polysaccharides promote immune cell activation by regulating the TCR/CD28 signaling pathway [10]; however, the differences in potency and specificity among various species remain poorly understood. Zebrafish serve as a suitable model for such investigations, given their 87% genomic similarity to humans and the conservation of over 80% of human disease-related proteins [11]. Vinorelbine (NVB)—a semi-synthetic vinca alkaloid—exerts broad-spectrum antitumor activity by inhibiting tubulin polymerization but significantly suppresses bone marrow hematopoietic function, thus reducing platelets, red/white blood cells (including neutrophils, lymphocytes, and T cells), and ultimately impairing body immunity [12,13]. In immunocompetence evaluation studies, various fluorescently labeled zebrafish models of immune cells such as neutrophils and macrophages have been established. Fluorescence microscopy can be used to capture images of the fluorescent cells, following which the immunological activities of drugs are evaluated by comparing the number or fluorescence intensity of these cells [14,15]. Therefore, creating an immunocompromised zebrafish model induced by NVB to comparatively evaluate the immunomodulatory effects of CP polysaccharides is expected to not only help clarify the mechanistic basis for their inter-varietal differences in efficacy, but also to support the optimized utilization of CP resources.

This study focused on WD and BTD, employing water extraction followed by alcohol precipitation to isolate polysaccharides from CP. This method is simple, low-cost, highly safe, and associated with minimal pollution. The crude polysaccharides obtained were subsequently purified, and structural characterization was performed using high-performance liquid chromatography (HPLC), gas chromatography–mass spectrometry (GC-MS), Fourier transform infrared spectroscopy (FT-IR), and nuclear magnetic resonance (NMR) techniques. Finally, an immunocompromised zebrafish model was established to evaluate the immunomodulatory activities of the two CP-derived polysaccharides through neutrophil counts, macrophage counts, and ELISA indicators. These findings provide a scientific basis for cultivar selection, quality evaluation, and the development of polysaccharide-based functional foods in CP species.

## 2. Materials and Methods

### 2.1. Reagents and Materials

The roots of two intraspecific varieties of *Codonopsis pilosula* (common name: Dangshen) were collected from Gansu Province, China, including *Codonopsis pilosula* Nannf. var. *modesta* (Nannf.) L.T.Shen (WD) and *Codonopsis pilosula* (Franch.) Nannf. (BTD). DEAE-52 cellulose chromatography columns, vincristine, and levamisole hydrochloride were purchased from Shanghai Yuanye Biotechnology Co., Ltd. (Shanghai, China). Monosaccharide standards and trifluoroacetic acid (TFA) were supplied by BoRui Saccharide Biotech Co., Ltd., Yangzhou, China). Zebrafish serum tumor necrosis factor-α (TNF-α), interleukin-1β (IL-1β), and interleukin-6 (IL-6) enzyme-linked immunosorbent assay (ELISA) kits were purchased from Shanghai Enzyme-Linked Bio-Technology Co., Ltd. (Shanghai, China). All other chemicals and reagents were of analytical grade.

### 2.2. Polysaccharide Extraction, Separation, and Purification

Crude polysaccharides from WD and BTD were extracted using the water extraction and ethanol precipitation method, according to a previously reported protocol [16] with minor modifications. Briefly, the roots of WD and BTD were dried at 50 °C, pulverized, and sieved (60 mesh). The powder was defatted three times in a constant temperature water bath (70 °C, 1 h per cycle) using 95% (*v*/*v*) ethanol at a solid-to-liquid ratio of 1:3 (*w*/*v*). The filter residue was then collected and air-dried. Subsequently, the residue was extracted twice under reflux at 80 °C with distilled water at a solid-to-liquid ratio of 1:20 (*w*/*v*). The combined supernatant was filtered and concentrated to one-quarter of its original volume using a rotary evaporator. Polysaccharides were precipitated by slowly adding anhydrous ethanol to a final concentration of 80% (*v*/*v*), and the mixture was stored overnight at 4 °C to facilitate precipitation. The precipitate was collected, dissolved in distilled water, and subjected to deproteinization using four volumes of Sevag reagent (chloroform/n-butanol = 4:1). The solution was shaken for 20 min, centrifuged at 8000 rpm for 20 min, and the supernatant was collected. This deproteinization process was repeated at least five times, until no free proteins were detected. The resulting supernatant was concentrated via rotary evaporation to remove residual organic solvents, dialyzed for 3 days with water changes every 4 h, and finally freeze dried to obtain the respective crude polysaccharides from WD and BTD.

The crude polysaccharides were separately loaded onto a DEAE-52 cellulose column (5 × 100 cm) for separation and purification [17]. Elution was performed starting with distilled water (0 M NaCl) followed by a linear gradient of 0 M to 0.5 M sodium chloride solution at a flow rate of 10 mL/min, and fractions were collected automatically (50 mL per tube). The collected fractions were quantified using the phenol–sulfuric acid method [18] and an absorbance–fraction number graph was plotted. As the water-eluted fraction exhibited the highest polysaccharide content, this fraction was dialyzed (molecular weight cut-off: 500 Da) and subsequently freeze dried to obtain the purified polysaccharides, denoted WCP and BCP. The final yield of each purified polysaccharide was calculated accordingly.

### 2.3. Structural Analysis of WCP and BCP

#### 2.3.1. Determination of Molecular Weight (Mw)

The molecular weights of WCP and BCP were determined using a multi-angle laser light scattering instrument (HPSEC-MALLS-RI, Wyatt Technology Corporation, Santa Barbara, CA, USA). Separation was performed on a Shodex OHpak SB-806M HQ (Shodex, Tokyo, Japan), column maintained at 35 °C, using 0.1 M NaCl as the mobile phase at a flow rate of 0.5 mL/min [19]. Standard dextran samples of different molecular weights were dissolved in ultrapure water to a concentration of 2 mg/mL, filtered through a 0.45 μm aqueous membrane, and injected at a volume of 100 μL. Similarly, 10 mg of WCP or BCP was dissolved in 5 mL of 0.1 M NaCl solution, filtered through a 0.45 μm aqueous membrane, and transferred to injection vials for chromatographic analysis.

#### 2.3.2. Fourier Transform Infrared Spectroscopy and Full-Wavelength UV Spectroscopy

Analysis was performed using a Fourier transform infrared spectrometer (TENSOR 27, Bruker Corporation, Denver, CO, USA) with the KBr pressing method [20]. The spectrum was scanned 60 times within the range of 4000 cm^−1^ to 400 cm^−1^ at a resolution of 4 cm^−1^. The samples were characterized by observing the absorption characteristic peaks in the infrared spectrum. Full-wavelength scans of WCP and BCP at a concentration of 1 mg/mL were performed using a UV spectrophotometer (Lambda35, PerkinElmer, Inc., Shelton, CT, USA) over the wavelength range of 190–500 nm [21].

#### 2.3.3. Monosaccharide Composition Analysis

Monosaccharide composition was determined using ion chromatography (IC). Briefly, 5 mg each of 11 monosaccharide standards (fucose, rhamnose, arabinose, galactose, glucose, xylose, mannose, fructose, ribose, galacturonic acid, glucuronic acid) was accurately weighed and hydrolyzed with 2 mL of 3 M trifluoroacetic acid (TFA) at 80 °C for 2 h. The hydrolyzed solutions were then transferred to clean tubes, dried under a nitrogen stream, and reconstituted in 5 mL of ultrapure water to obtain standard stock solutions after vortex mixing. Mixed standards at different concentration levels were prepared through precise dilution of individual stock solutions. Quantification of each monosaccharide was carried out using an absolute quantification approach, and molar ratios were calculated based on their respective molecular weights.

Approximately 5 mg of the sample was accurately weighed into an ampoule, then hydrolyzed with 2 mL of 3 M trifluoroacetic acid (TFA) at 80 °C for 2 h. The resulting hydrolysate was transferred to a clean tube, evaporated to dryness under a nitrogen stream, and reconstituted in 5 mL of deionized water. After thorough vortex mixing, a 100 µL aliquot was diluted with 900 µL of deionized water and centrifuged at 12,000 rpm for 5 min. The supernatant was collected for subsequent ion chromatography (IC) analysis.

#### 2.3.4. Methylation Assay

Approximately 2–3 mg of polysaccharide sample was weighed into a glass reaction flask. After adding 1 mL of anhydrous DMSO, Methylation Reagent A (anhydrous alkali solution) was rapidly introduced, and the flask was sealed and subjected to ultrasonication to ensure complete dissolution. Methylation Reagent B (iodomethane solution) was then added, and the reaction was allowed to proceed at 30 °C for 60 min in a magnetic stirring water bath. The reaction was terminated by adding 2 mL of ultrapure water. The resulting mixture was dialyzed using a 1000 Da molecular weight cut-off membrane for 24 h, followed by freeze drying. For FT-IR analysis, 2 mg of the dialyzed sample was precisely mixed with 200 mg of potassium bromide and pressed into pellets. Blank control pellets were prepared using pure potassium bromide powder. Fourier transform infrared spectroscopy was performed to verify complete methylation prior to further experimental steps.

The methylated polysaccharide was hydrolyzed with 1 mL of 2 M trifluoroacetic acid (TFA) at 100 °C for 90 min. After hydrolysis, the solution was evaporated to dryness using a rotary evaporator. The residue was dissolved in 2 mL of double-distilled water and reduced with 60 mg of sodium borohydride for 8 h. The mixture was then neutralized with glacial acetic acid, concentrated via rotary evaporation, and dried at 101 °C. Acetylation was performed by adding 1 mL of acetic anhydride and incubating at 100 °C for 1 h. After cooling, the reaction was quenched by adding 10 mL of pure water.

The acetylated product was dissolved in 3 mL of dichloromethane (CH_2_Cl_2_) and transferred to a separatory funnel. A small amount of distilled water was added, and the mixture was shaken thoroughly. The upper aqueous layer was removed, and this washing procedure was repeated four times. The organic layer was then dried over an appropriate amount of anhydrous sodium sulfate, concentrated to approximately 1 mL, and transferred to an HPLC vial. Analysis was performed using a Thermo Scientific 1300–7000 gas chromatography–mass spectrometry (GC-MS) system with the following conditions:

Column: HP-INNOWAX capillary column (30 m × 0.32 mm i.d., 0.25 μm film thickness); Temperature program: Initial temperature set at 140 °C, then ramped at 1 °C/min to 230 °C; Injection port temperature: 250 °C; Detector temperature: 250 °C; Carrier gas: Helium; Flow rate: 1 mL/min.

#### 2.3.5. Nuclear Magnetic Resonance Spectroscopy

Fifty milligrams each of WCP and BCP were separately dissolved in 0.5 mL of deuterated water (D_2_O). The solutions were centrifuged, and the resulting supernatants were transferred into standard 5 mm NMR tubes. All NMR spectra—including ^1^H NMR, ^13^C NMR, COSY, HSQC, and HMBC—were acquired at 700 MHz using a Bruker NMR spectrometer.

#### 2.3.6. Scanning Electron Microscopy

WCP and BCP powders were evenly spread on the sample stage to form a uniform layer, gently pressed for fixation, and subjected to gold coating. The prepared samples were then imaged using a Hitachi SU8010 instrument (Hitachi, Ltd., Tokyo, Japan) at various magnifications to examine their microstructural features.

#### 2.3.7. X-Ray Diffraction Analysis

An appropriate amount of WCP and BCP was placed at the center of a specimen slide, and spread evenly with a glass coverslip. The samples were then scanned on the X-ray diffractometer stage under the following conditions: 2θ range from 5° to 90°, scanning speed of 2°/min.

### 2.4. WCP and BCP Immunological Activity Studies

#### 2.4.1. Experimental Animals

Wild-type AB zebrafish and transgenic lines Tg(lyz:DsRed) (neutrophil-specific) and Tg(mpeg1:EGFP) (macrophage-specific) were obtained from the China Zebrafish Resource Center. Zebrafish were bred through natural pairing at 28 ± 0.5 °C, fed twice daily (morning and evening), and maintained under standard light/dark conditions (14 h/10 h). For natural spawning, male and female zebrafish were placed in breeding tanks at a 1:1 ratio the night before embryo collection. The following morning, embryos were collected, rinsed, and subsequently cultured at 28 °C in an incubator [22].

#### 2.4.2. Maximum Tolerable Dose Determination

At 3 days post-fertilization (3 dpf), 300 healthy zebrafish AB larvae were randomly selected and divided into a control group and WCP/BCP treatment groups. Treatment groups were further subdivided into 7 concentration groups (10 fish per group) with 3 parallel replicates at each concentration: 0.1, 0.4, 0.8, 1, 2, 3.2, and 4 mg/mL. After 24 h of exposure, the status of the zebrafish was observed and mortality at each concentration was recorded.

#### 2.4.3. Establishment of the NVB-Induced Immunodeficiency Model and Drug Administration

Healthy zebrafish larvae at 3 dpf were randomly selected and placed into 6-well plates at 15 larvae per well, with 3 replicates. Groups included a blank control, model group, positive drug group, and WCP/BCP treatment groups at varying concentrations. Each well received a total volume of 10 mL of the corresponding solution: the blank control group was maintained in embryonic water; the model group was exposed to 200 μg/mL NVB solution; the positive control group received 80 μg/mL Levamisole hydrochloride (LH) solution; and the treatment groups were administered WCP or BCP at specified concentrations. Except for the blank control, all other groups were subjected to a final modeling drug concentration of 200 μg/mL NVB. All groups were incubated at 28 °C under a 14 h/10 h light/dark cycle for the duration of the experiment.

#### 2.4.4. Neutrophil and Macrophage Counts

Neutrophils and macrophages represent two fundamental components of the immune system, playing indispensable roles in initiating immune defenses and regulating inflammation [23]. Both cell types can modulate the functional activities of other immune cells by secreting cytokines and chemokines, thereby achieving orderly coordination of immune responses [24]. Healthy 3 dpf transgenic larvae (Tg(lyz:DsRed) for neutrophils, Tg(mpeg1:EGFP) for macrophages) were treated according to the process outlined in Section 2.4.3 (3 independent biological replicates, 15 larvae per replicate). After 24 h of exposure, the larvae were anesthetized with 0.02% tricaine methanesulfonate (MS-222) and imaged using fluorescence microscopy. The acquired images were analyzed with the ImageJ software (fiji-win64) to quantify the fluorescence intensity of neutrophils and macrophages in the zebrafish. The numbers of neutrophils and macrophages in the tail were recorded for each group.

#### 2.4.5. Inflammatory Cytokine Content Assay

TNF-α, IL-1β, and IL-6 are key cytokines involved in immune regulation [25,26]. After 24 h of treatment (Section 2.4.3), 15 healthy 3 dpf zebrafish larvae (AB line, juvenile fish) per biological replicate were collected in 1.5 mL Eppendorf tubes, with 3 independent biological replicates set for each group. The juvenile fish were subjected to low-temperature shock treatment and soaked in an ice bath at 0–4 °C for 30 min. The treated larvae were washed 3 times with ddH_2_O, then homogenized in 1 mL of ice-cold 0.9% (*w*/*v*) physiological saline using a tissue cryomill for 3 min. The homogenate was centrifuged at 12,000 rpm for 15 min at 4 °C, and the supernatant was carefully collected for subsequent analysis. The total protein concentration in the supernatant was normalized using a BCA protein assay kit (Solarbio Science & Technology Co., Ltd., Beijing, China), according to the manufacturer’s instructions. Subsequently, the levels of TNF-α, IL-1β, and IL-6 in the supernatant were measured using enzyme-linked immunosorbent assay (ELISA) kits following the kit protocols.

#### 2.4.6. Statistical Analysis

All results are expressed as mean ± standard deviation. Statistical analyses were performed using the GraphPad Prism 9.5 and Origin 2024 software. Differences between groups were assessed via one-way ANOVA, with a *p*-value less than 0.05 considered to indicate statistical significance.

## 3. Results and Discussion

### 3.1. Extraction and Purification Analysis

**Figure 1:**
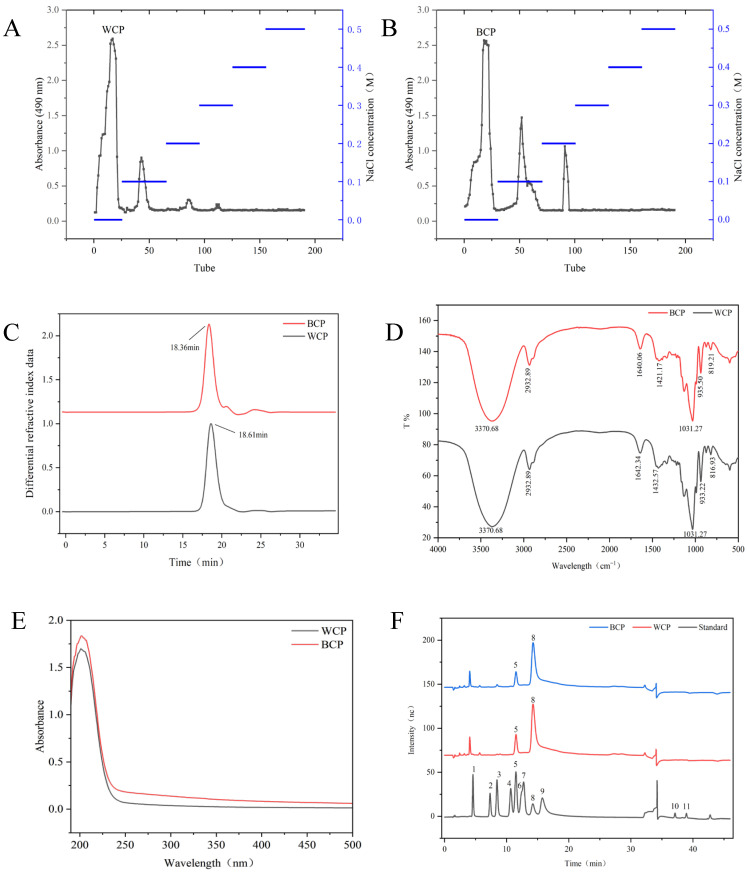
Isolation and structural characterization of WCP and BCP. (**A**) Elution curve of WCP on DEAE-52 cellulose column; (**B**) elution curve of BCP on DEAE-52 cellulose column; (**C**) molecular weight measurement using an 18-angle laser instrument; (**D**) FT-IR analysis of WCP and BCP; (**E**) UV-vis scanning spectrum; (**F**) monosaccharide composition analysis (1. Fuc; 2. Rha; 3. Ara; 4. Gal; 5. Glc; 6. Man; 7. Xyl; 8. Fru; 9. Rib; 10. GalA; 11. GlcA).

Polysaccharides were extracted from WD and BTD using the water extraction and ethanol precipitation method. Protein removal was achieved with Sevag reagent, followed by dialysis and freeze drying to obtain crude polysaccharides from WD and BTD. The yields were 10.63% and 5.18%, respectively, with crude polysaccharide purities of 66.67% and 73.80%. The purity of polysaccharides was determined using the phenol–sulfuric acid method. The absence of residual proteins in the treated samples was verified via the Coomassie brilliant blue G-250 method; in particular, no characteristic absorption at 595 nm was detected after the deproteinization process, confirming the effective removal of proteins. Further purification was carried out on DEAE-52 cellulose columns eluted with NaCl solutions at concentrations of 0, 0.1, 0.2, 0.3, 0.4, and 0.5 mol/L. The elution curves are presented in Figure 1A,B. Under 0 mol/L NaCl elution, distinct absorption peaks at 490 nm were observed for WCP and BCP, yielding 45% ± 2.43% and 32% ± 2.2%, respectively. Their purities of these fractions reached 93.96% ± 1.05% for WCP and 95.03% ± 0.83% for BCP, satisfying the criteria for further use. Notably, the water-eluted WCP and BCP align with most *Codonopsis* polysaccharides [27], consistent with the observation that neutral polysaccharides are the core bioactive fraction in this genus. The noticeable difference in yield and subtle variations in purity between WCP and BCP suggest that these two polysaccharide fractions may possess distinct structural features—a factor that may be associated with potential disparities in their bioactivity, thereby necessitating further detailed structural and functional characterization.

### 3.2. Molecular Weight Analysis

Molecular weight is a critical parameter for understanding the chemical and physical properties of polysaccharides. The uniformity of the molecular weight distribution affects the solubility, stability, and biological activity of a complex [28]. It has been reported that a lower Mw/Mn ratio in polysaccharides typically signifies a more uniform molecular weight distribution, and this favorable distribution characteristic has been associated with enhanced biological activity in some studies [29]. As shown in Figure 1C, the chromatograms of both WCP and BCP exhibited symmetrical single peaks. WCP had a relative molecular weight (Mw) of 6.444 kDa and an Mw/Mn ratio of 1.116; BCP had a relative molecular weight (Mw) of 15.71 kDa with Mw/Mn = 1.744. Compared to BCP, WCP exhibited a more homogeneous molecular weight distribution, which has been linked to superior water solubility and higher physicochemical stability in related polysaccharide research. In contrast, the broader molecular weight distribution of BCP is associated with a greater potential for variability in properties, fluctuations in functional performance, and possible loss of certain active components.

### 3.3. Fourier Transform Infrared Spectroscopy and Ultraviolet Spectroscopy

Fourier transform infrared spectroscopy (FTIR), as an analytical technique combining rapid detection with non-destructive properties, is capable of providing extensive structural information about substances and has been widely applied for the characterization of polysaccharides [30]. As shown in Figure 1D, the infrared spectra of WCP and BCP exhibited various characteristic absorption peaks typical of polysaccharides across the 4000–400 cm^−1^ range. A broad absorption peak around 3370 cm^−1^ corresponds to the O–H stretching vibration of sugar residues; the absorption peak at 2933 cm^−1^ originates from C-H stretching vibrations in the polysaccharide [31]; the peak near 1640 cm^−1^ is attributed to bending vibrations of bound water in the polysaccharide [32]; and the absorption around 1031 cm^−1^ corresponds to C-O stretching vibrations, indicating the presence of a pyranose ring [33]. However, differences exist in the peaks related to glycosidic bond configuration and the intensity of certain functional group peaks. The characteristic peak for the β-glycosidic bond at 935 cm^−1^ is stronger in BCP, while the characteristic peak for the α-glycosidic bond at 817 cm^−1^ is relatively prominent in WCP [34]. These spectral differences reflect structural variations between the two polysaccharides, such as variations in glycosidic bond ratios and hydrogen bond network density, which may be associated with their biological activities.

Ultraviolet spectroscopy serves as a core method for identifying proteins and nucleic acids in samples. The full-wavelength UV scans of both WCP and BCP revealed no absorption peaks at 260 nm or 280 nm, as depicted in Figure 1E, indicating the absence of nucleic acids and free proteins in the purified polysaccharide samples.

### 3.4. Monosaccharide Composition Analysis

Monosaccharide composition analysis is crucial for elucidating polysaccharide structures. Differences in monosaccharide types and contents have been linked to variations in the linkage types, bonding patterns, and spatial configuration of glycosidic bonds within polysaccharides, which are associated with differences in the biological activities of polysaccharides. The monosaccharide compositions of WCP and BCP were determined via ion chromatography through comparison with monosaccharide reference standards. As shown in Figure 1F, both polysaccharides consisted predominantly of fructose with a minor amount of glucose. Specifically, WCP contained 95.8% fructose and 4.2% glucose, while BCP contained 96.5% fructose and 3.5% glucose. These results are consistent with previous studies on *Codonopsis* polysaccharides [16], which reported that polysaccharides isolated from this genus are primarily composed of fructose with trace glucose. The subtle difference in the fructose-to-glucose ratio between WCP and BCP may be associated with variations in their glycosidic linkage patterns and chain architectures, which could potentially correlate with the divergence in their physicochemical properties and bioactivities.

### 3.5. Methylation Analysis

Methylation analysis serves as a cornerstone technique for polysaccharide structural characterization, enabling precise identification of glycosidic linkage types and accurate determination of molar ratios between different linkages [35]. As presented in Table 1, both WCP and BCP were found to share an inulin-type fructan core structure, primarily consisting of a →1)-Fruf-(2→ backbone. However, the molar ratio of key structural units exhibits notable varietal distinctions. In WCP, the →1)-Fruf-(2→ backbone unit accounted for 77.7%, while the branching unit →1,6)-Fruf-(2→ constituted only 0.6%. Neither →6)-Fruf-(2→ nor terminal Fruf-(2→ units were detected, exhibiting a “highly linear, low-branching” structural profile. In contrast, BCP showed a reduced proportion of →1)-Fruf-(2→ backbone units (69.6%), while the combined proportion of branching-related units →1,6) Fruf-(2→ 1.4% + →6) Fruf-(2→ 0.9%) reached 2.3%. Concurrently, the proportion of terminal residues (Fruf-(2→+Glcp-(1→) increased to 28.2%, forming a “slightly shorter backbone, highly branched” structure. These structural disparities may be potentially associated with varietal classification and growth conditions. Furthermore, they offer a basis for distinguishing the two CP varieties and provide clear directions for subsequent validation of potential variety–activity associations.

### 3.6. Nuclear Magnetic Resonance Analysis

First, the WCP spectra were analyzed. In polysaccharides, the number of aglycone residues and carbon atoms can be determined from ^1^H NMR and ^13^C NMR spectra. The ^13^C NMR spectrum of WCP exhibited characteristic signals consistent with a fructan structure; in particular, three carbon signals were observed in the low-field region at *δ*_C_ 103.63, 103.18, and 103.08. The absence of corresponding hydrogen signals in the HSQC spectrum indicated that these signals originate from the C-2 carbons of fructose residues. Based on the relevant literature and methylation results, *δ*_C_ 103.63, 103.18, and 103.08 were designated as A, B, and C, respectively (Table 2).

**Figure 2:**
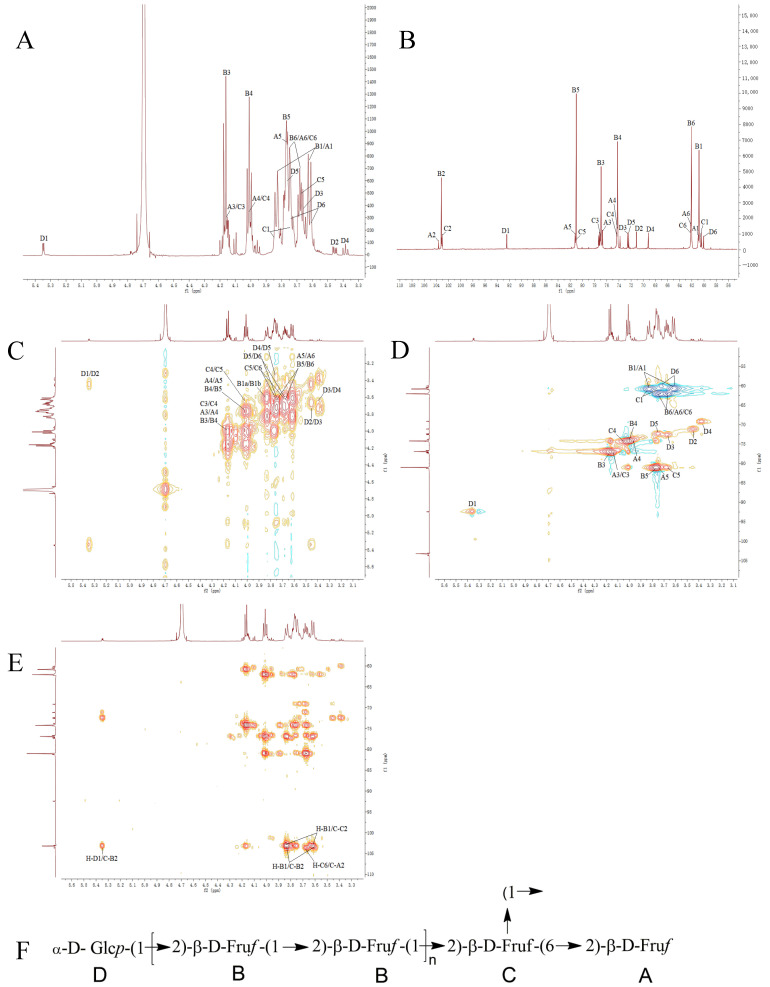
The NMR spectra of WCP. (**A**) ^1^H NMR spectrum; (**B**) ^13^C NMR spectrum; (**C**) ^1^H-^1^H COSY spectrum; (**D**) HSQC spectrum; (**E**) HMBC spectrum; (**F**) the proposed structure of WCP.

The H and C signals of the A, B, and C sugar residue fragments were identified from ^1^H NMR, ^13^C NMR, HSQC, HMBC, and ^1^H-^1^H COSY spectra (Figure 2). The C-1, C-3, C-4, C-5, and C-6 signals in fragment A were δ 60.97 (3.83, 3.62), 76.70 (4.16), 74.31 (4.00), 81.80 (3.76), and 62.19 (3.73, 3.68), respectively, indicating fragment A as β-D-Fruf-(2→; in fragment B, the C-1, C-3, C-4, C-5, and C-6 signals were δ 60.83 (3.83, 3.62), 76.92 (4.17), 74.21 (4.01), 81.01 (3.77), and 62.06 (3.73, 3.68), respectively, from which fragment B was inferred as →1)-β-D-Fruf-(2→; and the fragment C signals at C-1, C-3, C-4, C-5, C-6 were δ 60.65 (3.83, 3.75), 77.22 (4.16), 74.35 (4.00), 80.02 (3.68), and 62.22 (3.73, 3.68), respectively, indicating fragment C as →1,6)-β-D-Fruf-(2→ [36].

Based on the signals in the ^1^H, ^13^C NMR, and HSQC spectra, WCP displays an additional terminal signal at δ_H/C_ 5.35/92.42 (designated as fragment D) alongside its characteristic fructose resonances. The terminal signal of fragment D indicates its α-configuration. The H and C signals of the D-sugar residue fragment were determined from the ^1^H NMR, ^13^C NMR, HSQC, HMBC, and ^1^H-^1^H COSY spectra (Figure 2). The signals in fragment D were δ 71.13 (3.45), 72.54 (3.65), 69.17 (3.38), 72.38 (3.73), and 60.05 (3.72, 3.61), respectively, indicating the structure of fragment D as α-D-Glcp(1→ [37].

The structural fragment sequence of WCP was further determined from the HMBC spectrum, in which correlated signals between H-B1 and C-B2 were observed, confirming the presence of the →1)-β-D-Fruf-(2→1)-β-D-Fruf-(2→ structure; the correlation signal between H-B1 and C-C2 confirmed the presence of the →1,6)-β-D-Fruf-(2→1)-β-D-Fruf-(2→ structure in WCP; the correlation signal between H-C6 and C-A2 confirmed the presence of the β-D-Fruf-(2→6,1)-β-D-Fruf-(2→ structure; and the correlated signals between H-D1 and C-B2 confirmed the presence of the α-D-Glcp-(1→2)-β-D-Fruf-(1→ structure in the WCP structure. The structure of WCP, inferred from the methylation assay results and NMR analysis, is shown in Figure 2.

**Figure 3:**
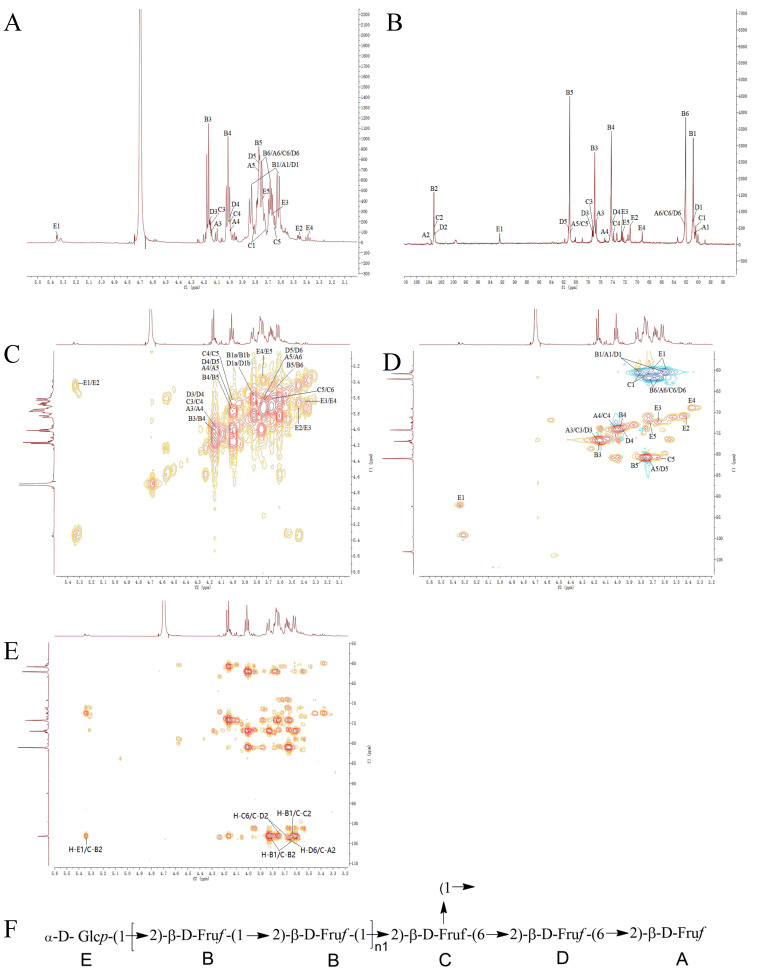
The NMR spectra of BCP. (**A**) ^1^H NMR spectrum; (**B**) ^13^C NMR spectrum; (**C**) ^1^H-^1^H COSY spectrum; (**D**) HSQC spectrum; (**E**) HMBC spectrum; (**F**) the proposed structure of BCP.

Next, the BCP spectra were analyzed. The ^13^C NMR spectrum of BCP exhibited characteristic signals of fructan, with four carbon signals in the low-field region at δC 103.63, 103.18, 103.09, and 103.03. The absence of corresponding hydrogen signals in the HSQC spectrum indicated that these carbon signals corresponded to the C-2 position in fructose. Based on the relevant literature and methylation results, δC 103.63, 103.18, 103.09, and 103.03 were designated as A, B, C, and D, respectively (Table 3). The H and C signals of the A, B, C, and D sugar residue fragments were identified through analysis of 1H NMR, 13C NMR, HSQC, HMBC, and 1H-1H COSY spectra (Figure 3). The C-1, C-3, C-4, C-5, and C-6 signals in fragment A were δ 60.43 (3.81, 3.61), 76.70 (4.15), 74.22 (4.02), 81.20 (3.75), and 62.08 (3.73, 3.66), respectively, indicating fragment A as β-D-Fruf-(2→; in fragment B, the C-1, C-3, C-4, C-5, and C-6 signals were δ 60.84 (3.81, 3.61), 76.93 (4.17), 74.22 (4.01), 81.03 (3.76), and 62.08 (3.73, 3.66), respectively, from which fragment B was inferred to be →1)-β-D-Fruf-(2→; fragment C showed signals at C-1, C-3, C-4, C-5, and C-6 as δ 60.07 (3.83, 3.71), 76.93 (4.15), 73.81 (4.02), 81.20 (3.67), and 62.08 (3.73, 3.66), respectively, indicating fragment C as →1,6)-β-D-Fruf-(2→; and, in fragment D, the signals for C-1, C-3, C-4, C-5, and C-6 were δ 60.97 (3.81, 3.61), 77.23 (4.16), 74.22 (4.01), 81.80 (3.76), and 62.08 (3.73, 3.66), respectively, from which fragment D was inferred to be →6)-β-D-Fruf-(2→ [36].

Based on the signals observed in the 1H, 13C NMR, and HSQC spectra, besides the fructose signal, BCP exhibited an additional terminal signal at δH/C 5.34/92.43, designated as fragment E. The terminal signal of fragment E indicates its α-configuration. The H and C signals of the E sugar residue fragment were determined from the 1H NMR, 13C NMR, HSQC, HMBC, and 1H-1H COSY spectra (Figure 3). The signals in fragment E were δ 71.14 (3.44), 72.39 (3.68), 69.17 (3.38), 72.17 (3.74), and 60.07 (3.71, 3.60), leading to the inference that fragment E is α-D-Glcp(1→ [37].

The structural fragment sequence of BCP was further determined from the HMBC spectrum, in which the correlated signals between H-B1 and C-B2 confirmed the presence of the →1)-β-D-Fruf-(2→1)-β-D-Fruf-(2→ structure in BCP; the correlation signal between H-B1 and C-C2 confirmed the presence of the →1,6)-β-D-Fruf-(2→1)-β-D-Fruf-(2→ structure in BCP; the correlation signal between H-D6 and C-A2 confirmed the presence of the β-D-Fruf-(2→6)-β-D-Fruf-(2→ structure in BCP; the correlated signal between H-C6 and C-D2 confirmed the presence of the →1,6)-β-D-Fruf-(2→6)-β-D-Fruf-(1,2→ structure in BCP; and the correlated signal between H-E1 and C-B2 confirms the presence of the α-D-Glcp-(1→2)-β-D-Fruf-(1→ structure in BCP. The structure of BCP, inferred from the methylation assay results and NMR analysis, is shown in Figure 3.

NMR analysis verified that WCP and BCP are fructans, in line with the structural features of neutral polysaccharides from the genus CP [16,38], characterized by the →1)-β-D-Fruf-(2→ backbone and α-D-Glcp-(1→ terminal residues. Notable intraspecific structural divergences were identified: WCP comprises three fructose-derived fragments and a linear-dominant, low-branching architecture (branching degree: 0.6%), while BCP exclusively harbors a previously unreported →6)-β-D-Fruf-(2→ fragment and a higher branching degree (2.3%). These structural differences correlate with the observed variations in molecular weight distribution between WCP and BCP, although potential associations with bioactivity disparities require further experimental validation. Moreover, the consistency between the NMR findings and methylation analysis enhances the rigor of the structural characterization performed in this study.

### 3.7. Scanning Electron Microscopy

**Figure 4:**
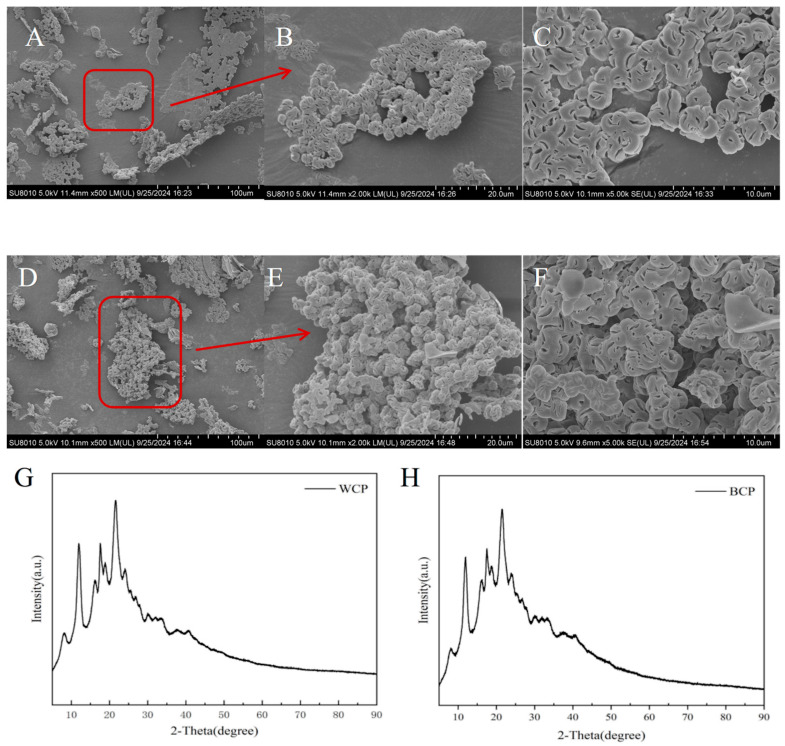
Scanning electron microscopy and XRD plots of WCP and BCP. (**A**) SEM image of WCP (×500); (**B**) SEM image of WCP (×2000); (**C**) SEM image of WCP (×5000); (**D**) SEM image of BCP (×500); (**E**) SEM image of BCP (×2000); (**F**) SEM image of BCP (×5000); (**G**) XRD pattern of WCP; (**H**) XRD pattern of BCP.

The microstructures of WCP and BCP were observed under scanning electron microscopy (SEM) at different field-of-view magnifications (×500, ×2000, and ×5000), as shown in Figure 4, and distinct differences were observed between the two polysaccharides. At 500× magnification (Figure 4A), WCP appeared in a dispersed state with localized agglomerations. Under higher magnifications (2000× and 5000×; Figure 4B,C), its surface exhibited a coarse, porous agglomerate structure with tightly bound particles. In contrast, BCP showed a more aggregated structure at 500× magnification (Figure 4D), which became increasingly prominent at 2000× (Figure 4E). At 5000× magnification (Figure 4F), the surface of BCP appeared denser and more complex, showing a stacked and consolidated architecture. These structural distinctions suggest divergent molecular packing behaviors between WCP and BCP, which have been linked to variations in intermolecular interactions [39]. The SEM results aligned with the NMR and methylation data: WCP’s linear structure correlates with its dispersed morphology, while BCP’s high branching matches its stacked packing and broad molecular weight distribution. These microstructural differences may affect immune cell receptor interactions, confirmation of which requires further functional tests.

### 3.8. X-Ray Diffraction Analysis

X-ray diffraction (XRD) is a commonly used technique for analyzing the crystal structure of materials. Partial scattering occurs when X-rays interact with a crystalline substance, with certain directions exhibiting enhanced intensity due to constructive interference. These diffraction profiles reflect the internal structural order of the material: the more regular the crystal structure, the sharper and more intense the diffraction peaks [39]. The crystalline or amorphous nature of a substance is significantly associated with key physicochemical properties such as its solubility, viscosity, and flexibility [40].

Analysis of the XRD patterns of WCP and BCP (Figure 4G,H) revealed that both polysaccharides exhibited characteristic diffraction peaks in the 2θ ≈ 12°, 17.5°, and 21.5° ranges, suggesting the presence of ordered crystalline regions, along with minor crystalline polysaccharides or polysaccharide complexes. The broadened nature of the observed peaks is consistent with the typical structural behavior of polysaccharides, in which semi-crystalline or amorphous domains often coexist with crystalline phases [41].

### 3.9. Immunological Activity Studies

Immunomodulatory activity is recognized as a core functional property of CP polysaccharides. Neutrophils and macrophages (key innate immune cells) and pro-inflammatory cytokines (TNF-α, IL-1β, IL-6) are pivotal indicators for evaluating immunomodulatory effects, and their dysregulation has been closely associated with immune dysfunction and persistent inflammation [25,42]. Therefore, we systematically evaluated the immunomodulatory potential of WCP and BCP in an NVB-induced immunocompromised zebrafish model, with reference to the experimental designs of related polysaccharide studies [25,26].

#### 3.9.1. Maximum Tolerated Concentration Determination

At WCP and BCP concentrations ranging from 0.1 to 2.0 mg/mL, no mortality or malformations were observed in the zebrafish, suggesting an absence of toxic effects within this dosage range (Table 4). These results suggest that WCP and BCP may be safely used in subsequent experiments at working concentrations of up to 2 mg/mL for immunomodulatory studies.

#### 3.9.2. Effects of WCP and BCP on Neutrophil and Macrophage Counts

Neutrophils and macrophages are recognized as foundational components of the innate immune system: neutrophils act as the “first line of defense” against pathogens by phagocytosing and eliminating invading microbes, while macrophages clear pathogens and apoptotic cells and regulate the adaptive immunity via antigen presentation [43,44]. Therefore, quantifying these cells is critical for assessing innate immune function recovery in immunocompromised models.

**Figure 5:**
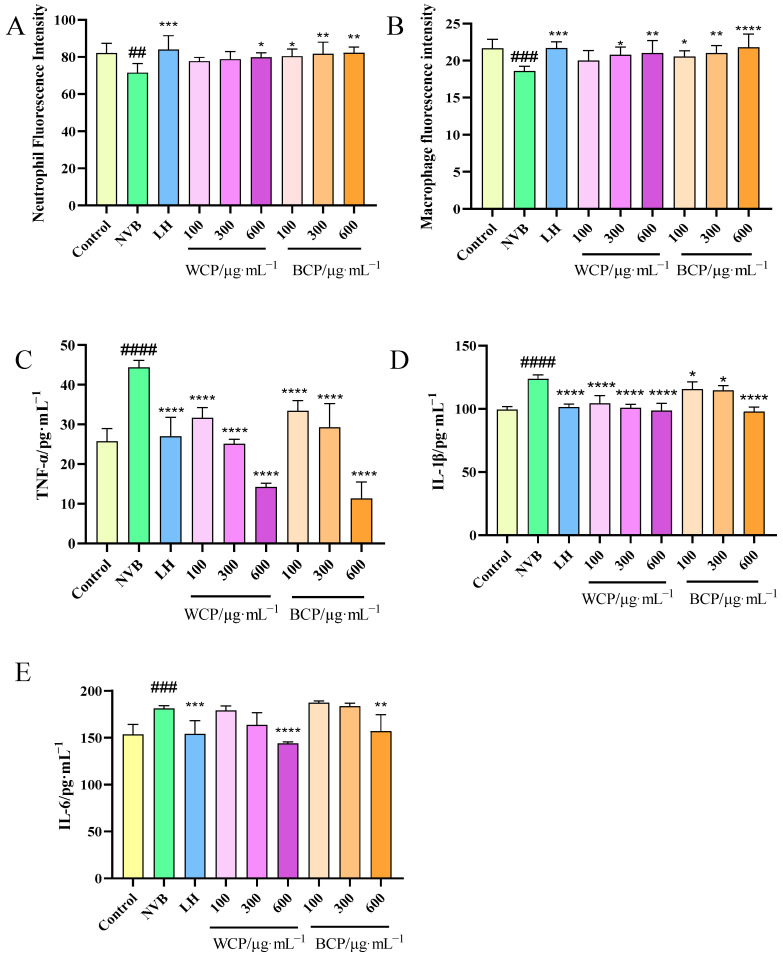
Effects of WCP and BCP on neutrophil fluorescence intensity, macrophage fluorescence intensity, and inflammatory factors in immunocompromised zebrafish. (**A**) Neutrophils; (**B**) macrophages (*n* = 8); (**C**) TNF-α levels; (**D**) IL-1β levels; (**E**) IL-6 levels (*n* = 30). Data are presented as the mean ± SD. ^##^ *p* < 0.01, ^###^ *p* < 0.001, ^####^ *p* < 0.0001 vs. control group; * *p* < 0.05, ** *p* < 0.01, *** *p* < 0.001, **** *p* < 0.0001 vs. NVB.

**Figure 6:**
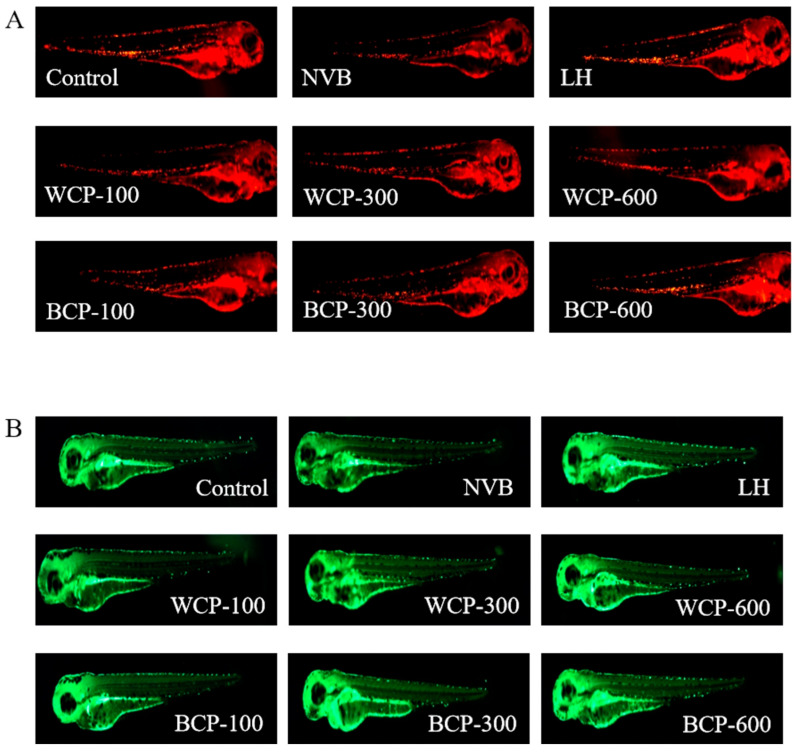
Fluorescence images of neutrophils and macrophages in zebrafish larvae after WCP and BCP treatment. (**A**) Neutrophil fluorescence images; (**B**) macrophage fluorescence images.

As shown in Figure 5A,B and Figure 6, compared with the blank control group, zebrafish in the model group exhibited significantly reduced neutrophil and macrophage counts. This indicates that NVB affected neutrophil and macrophage populations in the zebrafish—a change that is associated with the induction of immune impairment. In comparison to the model group, the fluorescence intensity of neutrophils and macrophages significantly increased in the WCP and BCP groups at different concentrations (100–600 μg/mL; *p* < 0.05). These results suggest that both polysaccharides may effectively promote restoration of the neutrophil and macrophage populations in the zebrafish tails, with a dose-dependent increase observed as polysaccharide concentration rises.

#### 3.9.3. Inflammatory Cytokine Levels

TNF-α, IL-1β, and IL-6 were selected as key indicators as they are recognized as major pro-inflammatory cytokines: TNF-α not only activates natural killer cells but also facilitates the maturation of dendritic cells, thereby boosting antigen-presenting capacity and efficiently linking the innate immunity to adaptive immune responses [45]; meanwhile, IL-1β and IL-6 foster the activation and differentiation of T cells and B cells, thereby augmenting cellular immunity and humoral immune responses [46]. In immunocompromised states, dysregulated signaling often triggers excessive secretion of these cytokines—a state linked to persistent inflammation and further immune impairment—and, thus, reducing their levels is critical for restoring immune homeostasis.

As shown in Figure 5C–E, compared with the blank control group, inflammatory cytokine levels in the model group increased to varying degrees after NVB modeling, indicating successful modeling. Compared with the model group, when WCP and BCP concentrations ranged from 100 to 600 μg/mL, TNF-α, IL-1β, and IL-6 levels were significantly reduced (*p* < 0.05). This observation is consistent with the findings of a recent study on *Saposhnikoviae Radix* polysaccharides, which exerted immunomodulatory effects by suppressing excessive pro-inflammatory cytokine secretion in NVB-induced zebrafish [25,26]. Collectively, these results suggest that WCP and BCP may effectively reduce inflammatory cytokine levels—a finding that supports their potential immunomodulatory activity.

As shown in Figure 5, BCP exhibited more prominent recovery effects on neutrophil and macrophage populations than WCP at equivalent concentrations, which may be associated with its higher branching degree and unique fragment, thus exposing more functional motifs to bind immune cell surface receptors and promote cell proliferation; meanwhile, WCP showed stronger inhibitory effects on pro-inflammatory cytokine secretion—a trait that may link to its linear, low-branching architecture and porous microstructure, which could potentially reduce steric hindrance, facilitate intracellular penetration, and efficiently block inflammatory signaling pathways. This divergent profile supports the notion of structure-dependent functional specificity of CP polysaccharides, where BCP prioritizes immune cell replenishment and WCP excels in inflammatory homeostasis regulation, laying a foundation for potential targeted application in immunomodulatory interventions.

These observations align with prior reports that CP polysaccharides exert analogous immunomodulatory effects in mouse models [47]. Among functionally analogous medicinal plants, *Saposhnikoviae Radix* polysaccharides (with galacturonic acid backbones) have also been shown to reduce pro-inflammatory cytokine levels in zebrafish [10,47]; however, WCP and BCP exhibited a more pronounced dose-dependent response, highlighting the structure-specific activity of fructan-type polysaccharides. Additionally, while CP polysaccharides have been documented to exhibit immunomodulatory activity [27], our study is the first to validate the in vivo immunomodulatory activities of variety-specific CP polysaccharides in a zebrafish model, addressing a critical knowledge gap in intraspecific CP polysaccharide research.

## 4. Conclusions

This study utilized a consistent water extraction and ethanol precipitation protocol to isolate polysaccharides from WD and BTD. Following separation and purification via DEAE-52 cellulose column chromatography, the homogeneous polysaccharides WCP and BCP were obtained and subsequently subjected to structural characterization and immunological activity evaluations. Structural characterization revealed molecular weights of 6.444 kDa and 15.71 kDa for WCP and BCP, respectively, which are primarily composed of abundant fructose and minor glucose residues. Methylation and NMR analyses indicated that both WCP and BCP are fructans. The glycosidic linkage pattern of WCP was identified as Fruf-(2→, Glcp-(1→,→1)-Fruf-(2→,→1,6)Fruf-(2→, while that of BCP consisted of Fruf-(2→, Glcp-(1→, →6)-Fruf-(2→, →1)-Fruf-(2→, →1,6)-Fruf-(2→. The surface morphology of the WCP and BCP exhibited significant differences. Additionally, they were found to exhibit certain immunostimulatory effects, significantly increasing the number of neutrophils and macrophages while reducing levels of TNF-α, IL-1β, and IL-6; notably, their immunomodulatory activities appear to be dose-dependent. To date, research on CP by scholars (both domestically and internationally) has primarily focused on the structural analysis of individual species, while comparative studies examining the structural variations among different CP polysaccharides remain relatively scarce. In conclusion, the findings of this study lay a theoretical basis for the development of CP-derived functional foods and cultivar selection. In future research, three-dimensional structural characterization techniques will be employed to resolve the spatial conformations of the polysaccharides, and their activities will be validated in mouse models, further clarifying their potential for clinical applications.

## Figures and Tables

**Table 1:** **Glycosidic** linkage analysis of WCP and BCP via methylation and GC-MS.

No.	Methylated Fragments	Major Fragment Mass	Glycosidic Bond Linkage Type	Mole Ratio	
				WCP	BCP
1	1,3,5,6-Me4-Manf/Glcf	87, 101, 129, 145, 161	Fruf-(2→	0.111	0.154
2	2,3,4,6-Me4-Glcp	43, 71, 87, 101, 117, 129, 145, 161, 205	Glcp-(1→	0.106	0.128
3	1,3,5-Me2-Manf/Glcf	99, 129, 145, 161, 189	→6)Fruf-(2→	0	0.009
4	3,5,6-Me3-Manf/Glcf	43, 71, 87, 99, 101, 129, 145, 161, 189	→1)-Fruf-(2→	0.777	0.696
5	3,5-Me2-Manf/Glcf	43, 87, 99, 129, 159, 189, 233	→1,6)Fruf-(2→	0.006	0.014

**Table 2:** ^1^H and ^13^C NMR chemical shift assignments for WCP.

Code	Glycosyl Residues		1	2	3	4	5	6
A	β-D-Fru*f*-(2→	H	3.83, 3.62		4.16	4.00	3.76	3.73, 3.68
		C	60.97	103.63	76.70	74.31	81.80	62.19
B	→1)-β-D-Fru*f*-(2→	H	3.83, 3.62		4.17	4.01	3.77	3.73, 3.68
		C	60.83	103.18	76.92	74.21	81.01	62.06
C	→1,6)-β-D-Fru*f*-(2→	H	3.83, 3.75		4.16	4.00	3.68	3.73, 3.68
		C	60.65	103.08	77.22	74.35	80.02	62.22
D	α-D- Glc*p*-(1→	H	5.35	3.45	3.65	3.38	3.73	3.72, 3.61
		C	92.42	71.13	72.54	69.17	72.38	60.05

**Table 3:** ^1^H and ^13^C NMR chemical shift assignments for BCP.

Code	Glycosyl Residues		1	2	3	4	5	6
A	β-D-Fru*f*-(2→	H	3.81, 3.61		4.15	4.02	3.75	3.73, 3.66
		C	60.43	103.63	76.70	74.22	81.20	62.08
B	→1)-β-D-Fru*f*-(2→	H	3.81, 3.61		4.17	4.01	3.76	3.73, 3.66
		C	60.84	103.18	76.93	74.22	81.03	62.08
C	→1,6)-β-D-Fru*f*-(2→	H	3.83, 3.71		4.15	4.02	3.67	3.73, 3.66
		C	60.07	103.09	76.93	73.81	81.20	62.08
D	→6)-β-D-Fru*f*-(2→	H	3.81, 3.61		4.16	4.01	3.76	3.73, 3.66
		C	60.97	103.03	77.23	74.22	81.80	62.08
E	α-D- Glc*p*-(1→	H	5.34	3.44	3.68	3.38	3.74	3.71, 3.60
		C	92.43	71.14	72.39	69.17	72.17	60.07

**Table 4:** Maximum tolerable dose of WCP and BCP in zebrafish larvae.

Concentration (mg/mL)	WCP Mortality (%)	BCP Mortality (%)
0.1	0	0
0.4	0	0
0.8	0	0
1.0	0	0
2.0	0	0
3.2	20	10
4.0	50	30

## Data Availability

The original contributions presented in the study are included in the article, further inquiries can be directed to the corresponding authors.

## Abbreviations

Abbreviation	Full Form
CP	*Codonopsis pilosula*
WD	*Codonopsis pilosula* Nannf. var. *modesta* (Nannf.) L.T.Shen
BTD	*Codonopsis pilosula* (Franch.) Nannf.
WCP	*Codonopsis pilosula* Nannf. var. *modesta* (Nannf.) L.T.Shen pure polysaccharides
BCP	*Codonopsis pilosula* (Franch.) Nannf pure polysaccharides
TFA	Trifluoroacetic acid
Mw	Molecular weight
Fuc	Fucose
Rha	Rhamnose
Ara	Arabinose
Gal	Galactose
Glc	Glucose
Man	Mannose
Xyl	Xylose
Fru	Fructose
Rib	Ribose
GalA	Galacturonic acid
GlcA	Gluconic acid
FT-IR	Fourier transform infrared
UV	Ultraviolet–Visible
SEM	Scanning electron microscope
XRD	X-Ray Diffractometer
NVB	Vinorelbine
LH	Levamisole hydrochloride
